# Recruitment and retention of older adults in Assisted Living Facilities to a clinical trial using technology for falls prevention: A qualitative case study of barriers and facilitators

**DOI:** 10.1111/hsc.13170

**Published:** 2020-09-11

**Authors:** Wytske M. A. Meekes, Claire Ford, Emma K. Stanmore

**Affiliations:** ^1^ Tranzo, Tilburg School of Social and Behavioural Sciences Tilburg University Tilburg Netherlands; ^2^ School of Health Sciences, Jean McFarlane Building The University of Manchester Manchester UK; ^3^ Manchester Academic Health Science Centre, Core Technology Facility The University of Manchester Manchester UK

**Keywords:** exercise/physical activity, falls prevention, older adults, recruitment and retention, technology

## Abstract

Older adults often have health complexities and higher levels of attrition. Even though they are the main users of healthcare, they are often not included in health research because the health research may not be well designed to accommodate their evolving health needs. One research area in which participation of older adults is essential focuses on improving physical function. In this field, there are many innovations and new technologies developed. Barriers and facilitators to recruit older adults to research that improves physical function by using technology are not well explored yet. This study aims to explore barriers and facilitators regarding recruitment and retention of older adults living in Assisted Living Facilities to a randomised controlled trial study that aimed to improve physical function by using technology. Nine semi‐structured interviews were conducted with four Scheme Managers, three therapists and two researchers. The interviews were transcribed. After open, axial and selective coding, the codes were thematic analysed in ATLAS.ti. Scheme Managers, therapists, researchers and older adults’ peers appear to play an important role in the recruitment and retention of older adults living in Assisted Living Facilities. Additionally, the technology itself and the presentation of the research appear to influence recruitment. Creating a social setting, inviting people face‐to‐face, demonstrating the technology, showing the benefits by presenting results from a pilot study and alleviating people's fears were experienced as important factors for recruitment. The results from this study can help other researcher to improve recruitment and retention strategies so evidence‐based practice in care for older adults can be improved to enhance quality of life of older adults.

**What is known**

- Older adults often have health complexities and higher levels of attrition when involved in health research.
- Barriers and facilitators to recruit older adults to research that improve physical function by using technology are not well explored yet.

**What is added**

- Scheme Managers, therapists, researchers and adult's peers can play an important role in the recruitment and retention of older adults living in Assisted Living Facilities into health research that aims to improve physical function by using technology.
- Inviting people face‐to‐face, demonstrating the technology during a real‐life session, showing the benefits by presenting results from a feasibility study and alleviating people's fears can positively influence recruitment.

## 1 INTRODUCTION

The proportion of older adults is increasing worldwide (WHO, 2018). Older adults frequently have comorbid conditions and require more health services compared to children and adults (Divo, Martinez, & Mannino, 2014; Vegda et al., 2009). Therefore, it is important to focus on this age group in health research to improve physical function through exercise and to promote healthy ageing (Booth, Roberts, & Laye, 2012; Kendrick et al., 2018). The main purpose of health research is to gather evidence for clinical practice, thus, a study population should represent the main users. Yet, despite this, older adults are often not included in health research because studies are often not well designed to accommodate the evolving health of older adults who may have health complexities and who experience higher levels of attrition (McMurdo et al., 2011; Ridda, MacIntyre, Lindley, & Tan, 2010). Furthermore, their various medical histories may cause heterogeneity in treatment response (Mody et al., 2008) leading to less conclusive results. Also, recruitment goals may not be reached due to high attrition. Carlisle, Kimmelman, Ramsay, and MacKinnon (2015) concluded in their study about trial recruitment that, in general, 19% of the trials closed due to recruitment problems or with less than 85% of the expected enrolment, compromising the statistical power of a study (Carlisle et al., 2015). Not meeting recruitment goals can have a negative impact on scientific, ethical, political and financial outcomes (Huang et al., 2018). More importantly, it negatively influences the outcome of trials that aim to improve healthcare for patients. A successful trial depends on successful recruitment and retention of participants (Wylie et al., 2015). Still, many studies experience barriers when recruiting older adults to research and to keep them engaged.

One research area in which participation of older adults is essential is falls prevention. Many falls prevention studies focus on improving the physical function of older adults. However, motivating older adults to start and complete interventions that improve physical function is experienced as difficult, in research as well as in daily practice (Laventure & Skelton, 2007; Van Ruiten, 2013). Additionally, technologies (e.g. Exergames, fall detection systems) are more often used as an effective means for older adults to stay healthy and independent, although not widely adopted or used by older adults (Coughlin, 2010; Lee & Coughlin, 2015). However, attrition rates can be high for technology‐based intervention studies such as those involving web‐based interventions (Peels et al., 2012) or wearable technology (Attig & Franke, 2020). Previous studies mention that factors, such as improved health, technological complexity, social interaction and lack of knowledge and confidence, influence older people's attitudes towards technology (Andrews, Brown, Hawley, & Astell, 2019; Barg‐Walkow, Harrington, Mitzner, Hartley, & Rogers, 2017; Vaportzis, Giatsi Clausen, & Gow, 2017). According to Lee and Coughlin (2015), certain factors are important to consider during designing, developing and delivering a technology as they influence adaptation and use (Lee & Coughlin, 2015). Consequently, these factors will also influence recruitment and retention to studies that use technology. Barriers and facilitators concerning intervention adherence that improve physical function have been investigated (Essery, Geraghty, Kirby, & Yardley, 2017; Jack, McLean, Moffett, & Gardiner, 2010; Picorelli, Pereira, Pereira, Felício, & Sherrington, 2014; Resnick et al., 2008). However, barriers and facilitators to recruit older adults to research that improves physical function by using technology are not well explored yet. In particular, there is a lack of studies in Assisted Living Facilities (also known as sheltered housing or extra care housing) in which residents retain their independence, living in flats or bungalows but can access care for activities of daily living (e.g. dressing, shopping). Residents living in Assisted Living Facilities tend to have more disabilities or degrees of frailty (according to Rockwood's definition (Clegg, Young, Iliffe, Rikkert, & Rockwood, 2013)) than community‐dwelling older adults, but are usually more independent than those who live in care homes. Therefore, the aim of this study is to explore the barriers and facilitators regarding recruitment and retention of older adults living in Assisted Living Facilities to health‐promoting research that aims to improve physical function by using technology.

## 2 METHODS

This qualitative case study took place as a sub‐study of a larger RCT study that aimed to explore the effectiveness of a suite of Exergames to improve balance and other outcomes in older adults in Assisted Living Facilities (Stanmore et al., 2019). Exergames are active video games that combine game play with physical activity. The Exergames used in this study are developed with older adults, academics and two falls prevention teams to improve the physical function and reduce fall risk of older adults. The Exergames are composed of evidence‐based OTAGO and FaME strength and balance exercises (Campbell et al., 1997; Skelton, Dinan, Campbell, & Rutherford, 2005). The Exergame system uses a Microsoft Kinect, an off‐the‐shelf 3D camera, to track the participant's movements. More information about this RCT, the inclusion and exclusion criteria of the participants and the Exergames can be found elsewhere (Stanmore et al., 2019).

In this qualitative case study, nine semi‐structured interviews were conducted at the end of the RCT study. All researchers, therapists and Scheme managers (SMs) from the intervention group of the RCT study were invited to participate if they were located in Greater Manchester due to their involvement in the recruitment process and observation of the study (i.e. explaining the purpose of the study, obtaining informed written consent from the participants). A topic guide was used to aid the semi‐structured interviews, which aimed to explore the barriers and facilitators of the recruitment and retention of participants in the RCT study. Questions were centred around the roles, characteristics of the therapists or SMs; dynamics within Assisted Living Facilities; relationships among managers, therapists and participants; and the reasons for Exergame attendance, non‐attendance and/or withdrawal from the study. The interviews were conducted by a research assistant who was not involved in the execution of the RCT study regarding the effectiveness of Exergames. The interviews were audio‐recorded and each interview took approximately 45 min. The interviews with the SMs were conducted in their offices and the interviews with the therapists and researchers were conducted in quiet rooms at the University of Manchester.

### 2.1 Setting

As described above, this study took place as a sub‐study of a larger RCT study (Stanmore et al., 2019). The RCT study was carried out at Assisted Living Facilities in the UK. Each facility was managed by a SM, whose role was to manage the Assisted Living Facility and support residents in a variety of ways (through accessing services and resources, and calling for help in an emergency situation). Before recruitment of residents, the researchers first approached the Housing Association Manager for approval to approach the SMs. After approval, the Housing Association Manager sent an email to all the SMs to inform them that they might be approached by the researchers. Subsequently the researchers invited the SMs of all the facilities to take part in the study. If interested, the researchers made an appointment with the SMs to explain the study procedures and to demonstrate the Exergames and the results of a previously conducted pilot study (Stanmore, Todd, & Skelton, 2015).

After the SM gave approval, the researchers invited all residents of the facility to a presentation about the Exergames in the communal lounge by distributing flyers to all flats in the facility. Interested residents came to the presentation and were invited to participate in the study. The study processes were explained, a demonstration of the Exergames was given and potential participants were given the opportunity to ask questions about the study. Older adults aged ≥ 55 were recruited from 18 Assisted Living Facilities in the UK. They were invited to play the Exergames three times a week during a period of 12 consecutive weeks (and assessed at baseline and 12 weeks) under supervision of a physiotherapist or physiotherapist assistant in the communal room of their facility. The duration of the Exergame session was set by the physiotherapist according to their assessment of the level of ability of the older adults and the time varied between 10 and 20 min.

### 2.2 Data analysis

After transcription, the interviews were open coded by two researchers (WM, ES). The codes were discussed and a coding scheme was developed from the different themes that emerged. The interviews were selective coded by two researchers (WM, ES) and compared. After consensus was reached, the coding was thematic analysed using ATLAS.ti 8 coding program (Friese, 2019).

### 2.3 Theoretical Framework

The coding scheme used for the analysis of the results was informed by the Health Belief Model (HBM) which was initially developed by psychologists of the Public Health Service in the USA. (Champion & Skinner, 2008; Hochbaum, 1958; Rosenstock, 1974). The aim of the HBM is to predict why people take action to prevent, screen or control health issues. The HBM was used in this study to be able to explain and understand the factors from the perspectives of the SMs, therapists and researchers that influence the likelihood of the older adults in the Assisted Living Facilities to engage in health‐promoting research using technology. The concepts of the HBM are operationalised in Table 1. The explored factors that influence the recruitment and retention of the older adults are related mainly to the concepts, Perceived Benefits versus. Perceived Barriers and Cues to Action of the HBM. Appendix 1 provides an overview of the codes used in this study and to which concepts of the HBM they relate.

**TABLE 1:** Health Belief Model concepts operationalised (Rosenstock, 1974)

Concept	Refers to
Modifying variables	Participant's characteristics
Perceived Seriousness	Assessment of how serious the fall risk is and the consequences of a fall
Perceived Susceptibility	Assessment of how high the fall risk is
Perceived Threat	The motivation to take part in the study and undertake the Exergames as a result of the perceived seriousness and susceptibility
Perceived Benefits	The beliefs that the Exergames will reduce the risk of falling and prevent severe injuries and increase independence
Perceived Barriers	Any perceived obstacles to engage in the Exergames
Self‐efficacy	Perception of having the competence to play the Exergames
Cues to Action	Internal or external triggers to play the Exergames
Likelihood of engaging in health‐promoting behaviour	The behaviour during participation in the research study and playing the Exergames

## 3 FINDINGS

In this study, nine semi‐structured interviews were conducted with four SMs of four Assisted Living Facilities in which the Exergames were conducted; one physiotherapist and two physiotherapist assistants who supervised the older adults during the Exergame intervention; and one senior researcher and one junior researcher who investigated the effectiveness of the Exergames. Participant characteristics are presented in Table 2.

**TABLE 2:** Participant characteristics

Name^a^	Gender	Function	Experience
Charles	M	Scheme Manager	10.5 years experience as Scheme Manager at participating Assisted Living Facility
Catherine	F	Scheme Manager	6 years experience as Scheme Manager at participating Assisted Living Facility
Charlotte	F	Scheme Manager	11 years experience as Scheme Manager at participating Assisted Living Facility
Collins	M	Scheme Manager	9 months experience as Scheme Manager at participating Assisted Living Facility
Anne	F	Physiotherapist	7 years experience as physiotherapist
Elinor	F	Physiotherapist assistant	2 years experience with falls prevention (also as coordinator)
Marianne	F	Physiotherapist assistant	3.5 years experience as physiotherapist assistant
Jane	F	Senior researcher	20 years experience with falls prevention research
Elizabeth	F	Junior researcher	1.5 years experience with falls prevention research

^a^ Names are pseudonyms.

According to the results of this study, the following persons appeared to influence the recruitment and retention of the older adults: SMs, therapists, researchers and older adults’ peers. Additionally, participants’ health, Exergame product itself and the presentation of the research appeared to have influence. Below the perceived barriers and facilitators are elaborated and an overview is presented in Table 3.

**TABLE 3:** Barriers and facilitators for recruitment and retention of older adults in Assisted Living Facilities

	Barriers	Facilitators
Scheme Managers	Busy/Lack of staff at facilities	SM helps to contact and approach residents
	Miscommunication or lack of communication	SM helps to distribute flyers and information about the study
		SM motivates residents to participate by praising and encouraging them
		SM provides notices and information about study
		SM’s presence during presentation about research project for resident
		Specific characteristics of SM such as non‐patronising, positive energy (see paragraph ‘Scheme Manager’)
Therapists	Preconceptions regarding older people	Therapist supervision of resident undertaking the Exergames
		Therapist advice regarding physical function
		Therapist maintaining a comfortable atmosphere in the group
		Therapist motivating residents by supporting them to feel confident and safe
		Therapist wearing physiotherapist uniforms
		Therapist explains how exercises may assist daily activities of living
		Gentle and non‐patronising approach
		Specific characteristics of therapist such as approachable, supportive (see paragraph ‘Therapists’)
Researchers	Patient information letter (according to ethical requirements)	Support from Housing Association Manager
	Assessment of cognitive function among residents with cognitive difficulties	Researchers’ presentation about the study by inviting residents face‐to‐face, demonstrating the Exergames and showing the benefits from a previous feasibility study.
		Government funded study and conducted by University (perceived prestige)
		Researcher's characteristic of being approachable
Peers	Tensions between residents/uncomfortable atmosphere	Peers cheering and clapping for each other
	Peer pressure to withdraw or to not participate	Relaxed and comfortable atmosphere
		Peers sharing positive experiences
		Having residents with strong personalities at the facility participating in the study
General	Comorbid health issues	Exergames providing feedback about performance
	Technical difficulties with the Exergames	
	Terminology in advertisement (‘exercise’)	
	Timing of the study (during summer many residents are on holiday)	
	Other activities at the facility (simultaneously)	

Abbreviation: SM, Scheme Manager.

### 3.1 Scheme managers

All participants acknowledged the influence of the SM on the recruitment and retention of the older adults into the Exergames study. The SM in many instances acted as a facilitator and often helped with contacting and approaching residents as they thought that the Exergames could improve their quality of life and reduce their risk of falls.

“the wardens (Scheme Managers) were helpful in some of the places, … and (they) helped us approach them (the residents).” – Anne, Physiotherapist

Some SMs also helped the research team with the recruitment by distributing flyers and informing residents about the Exergame study and reminding them about the dates and times of the presentations and sessions. These SMs also tried to motivate residents to participate in the Exergames by praising and encouraging them to play the games.

“But I’m a good motivator, I can get people, can say, I can really get to people and say come on, you'll be great,” – Catherine, Scheme Manager

“I would sort of monitor them from that point of view and also like [to give] praises. So when I go and see them do it, you make comments. I know a lot of their illnesses so when I see them raising their arms or legs or whatever it might be, I can tell straight away a massive improvement. So yeah, I think the encouragement. I think it's good to have that positiveness and let them see it, let them feel it. I think that might have helped contribute to some of the support they went through. So I think the managers play a part but it's a team thing.” – Charles, Scheme Manager.

The participants in this study also mentioned that the more engaged SMs prompted their residents about presentations or sessions. This was necessary according to the SMs because residents would otherwise forget. For example, one SM mentioned that notices should not be given too far in advance because residents would forget. In addition, the therapists experienced that if a SM was more involved and appreciated the potential benefits of the study, the group of residents participating in the study was bigger.

“But she (Scheme Manager) was very much wanting the residents to get involved, and I think that's why it was a bigger group anyway, because of her input. And the smaller group the scheme manager she was very… I think she was tied up in her office.” – Marianne, Physiotherapist assistant.

Some participants reported that if SMs were very busy and had to cover more than one facility due to lack of staff, they were perceived to be less approachable for residents and researchers and they had less time to get involved with or promote the study. Furthermore, clear and effective communication between the SMs and researchers appears to be essential for recruitment as miscommunication resulted in almost no attendees for a presentation. Also, the presence of a SM during a presentation was perceived by the researchers as a facilitator for recruitment. For example, when a SM was on holiday during a presentation, residents were much less likely to participate.

According to the therapists, the influence of the SM also depended on the level of health of the residents in a facility. They experienced that if the residents were more dependent (i.e. poorer mobility, comorbidities and more need for support with activities of daily living), they appeared to depend more on the SM and so the SM had a greater influence compared to facilities in which residents were more independent.

The participants of this study perceived that the following characteristics of the SM were facilitators for successful recruitment and retention: passionate about working with older adults; smiling; happy; positive energy; approachable; talking the same language as the residents; non‐patronising; encouraging residents; hands‐on relationship with the residents; able to motivate residents; being organised and knowing what is going on in the facility; caring; empathetic; positive; helpful; giving the residents confidence and praising the residents.

### 3.2 Therapists

The participants of this study acknowledged the influence of the physiotherapist and the two physio‐assistants on the retention of residents to the study. Below, the physiotherapist and physio‐assistants are referred to as therapists. Besides being able to provide the Exergames and give advice regarding physical function, other characteristics of the therapists also appear to be important. To maintain a comfortable atmosphere in a group, the therapists also needed to deal with tensions between some residents. The therapists had to be able to deal with the different personalities of the residents.

“He (a resident) can be quite scary. He has got Bipolar and I think he screamed and shouted at her (therapist) – I don't know if she was late or whatever but I was in the background but he was very much, you know, and he stormed back off to his flat and she was able to calm him down, and explained what had happened and he was fine then” – Catherine, Scheme Manager.

One therapist experienced that her own preconception about older adults could be a barrier for the execution of her work and that an open attitude towards older adults is important even if you have worked with older adults in the past.

“yeah they are very different. I think that this has been the biggest thing for me. Because I have been used to delivering exercise classes and I have worked in the community. So when you bring people together from the community they are very appreciative of going somewhere because they are lonely and I think that when you work in a scheme [Assisted Living Facility], for example the two schemes that I’ve been working with… this is their home. So you are going into their home, when you are going into the lounge and in that you have got all different dynamics and personalities that live in a home. So it is a bit like a family in a way, where you get conflicts within families with you know siblings. It is a bit like that really which I was not really prepared for” – Elinor, Physiotherapist assistant.

Therapists who were able to motivate older adults and let them feel confident and safe were experienced as important facilitators for the retention. One therapist mentioned that she wore physiotherapist uniforms, which might have increased the perception of safety among older adults. In addition, explaining how certain movements assist in activities of daily living (e.g. dressing or shopping) might have increased the motivation for continuing to take part in the study. Additionally, a gentle and non‐patronising approach was experienced as important.

“Sometimes when they are elderly, people talk to them like they are children or they are different. So I just speak to them like I am speaking to you now and that is, a lot of feedback that I got from the group saying “you treating us normally”, well you are! Why would not I? You know? But not everybody does. They start talking to us like (in loud, patronizing tone) “Do you want to do the exercise?” You know, because we may not be able to hear or see properly they treat us like we are daft.” – Elinor, Physiotherapist assistant.

Characteristics of the therapists that were experienced by the participants of this study as important were being approachable, enthusiastic, passionate and supportive.

### 3.3 Researchers

According to the results from this study, the researchers appear to have more influence on recruitment of the older adults in the Exergame study than on retention. The researchers explained that they first approached the Housing Association Manager for approval to approach the SMs. Some of the SMs mentioned that participating in the Exergame study was promoted by their Housing Association organisation. According to the researchers, having the Housing Association Manager promoting the Exergame study made recruitment of facilities easier.

“And the trust (Housing Association organization) they wanted to promote it, the bigger bosses and that. They wanted us to get involved in it, the Scheme Managers to push, well not to push but to ask them.” – Charlotte, Scheme Manager.

During a first appointment with a SM, the researchers presented the Exergames and explained the purpose and procedures of the study. When the SM gave approval, the researchers also presented the Exergames to the residents during a group presentation in the communal lounge of the facility. According to the researchers and some of the SMs, the presentations had a positive influence on the recruitment. Inviting people face‐to‐face, demonstrating the Exergames during a real‐life session, showing the benefits by presenting results from a pilot study and alleviating people's fears (e.g. regarding use of the technology or ability to join with certain health issues) were experienced as important factors for recruitment. According to one SM, the study being government funded and the study being conducted by the University helped recruitment as well as the study was deemed important and purposeful. Furthermore, the researchers mentioned that they received feedback from the older adults that they were approachable, which might have helped with the retention of older adults. In contrast, the patient information letter was experienced as a barrier and was perceived as not being user‐friendly because it has to meet several criteria for ethical approval.

“All this ethics information sheets, what they have to comply with, it's very off‐putting. They [participants] do not want to have to read three pages in detail as you can imagine.” – Jane, Researcher.

Furthermore, conducting assessments about cognitive function, such as the Addenbrooks Cognitive Examination III (Hsieh, Schubert, Hoon, Mioshi, & Hodges, 2013), were experienced as potential barriers for recruitment and retention for older adults with cognitive issues. These questionnaires were uncomfortable for some older adults as they were aware that they had some cognitive difficulties and the trained researchers needed to offer lots of reassurance and support during these assessments.

### 3.4 Peers

According to the participants of this study, the peer relationships of the older adults participating in the Exergames study were experienced to be both barriers and facilitators in the recruitment and retention. Tensions between residents on occasions resulted in an uncomfortable atmosphere at the facility. The SMs mentioned that these tensions might arise due to different personalities but could also be caused due to health issues (e.g. psychological disorders, cognitive decline).

“Eventually it ironed out issues and problems because you are dealing with lots of residents and some of them do not talk to each other for whatever reasons, just personality.” – Charles, Scheme Manager.

“Some people were very competitive, some in a helpful way, cheering and clapping and some not in a helpful way, perhaps wanting to be better than the next person. And just personalities and you do find that cognitive decline that some people, they have not got that filter where they just say things that perhaps they have not thought through or can be a bit… yeah less tactful.” – Jane, Senior researcher.

One SM also mentioned that if friends withdrew or were not interested, the remaining residents were more likely to withdraw as well. This was also noticed by the junior researcher at other facilities. Nevertheless, most of the time, peers were experienced as facilitators for the recruitment and retention in the Exergame study. Peers cheered and clapped for each other.

“You know more sort of cheering each other on and staying for the duration of the game...”‐ Marianne, Physiotherapist assistant

At most facilities, the atmosphere was experienced by therapists and researchers as relaxed and comfortable which made the execution of the Exergames more pleasant. Peers would also mention their positive experiences with the Exergames which may have motivated others to join in or to keep on going. Also, being able to get the residents with a strong personality (i.e. influential) on board with the study may have made recruitment of other residents easier.

### 3.5 General

The health of the older adults, the Exergame product and the set‐up of the study could have influenced recruitment and retention according to the participants. Many of the older adults who participated in the Exergame study had multiple health issues such as osteoarthritis, chronic obstructive pulmonary disease and diabetes. Therefore, they had regular hospital visits and admissions or due to acute illness were not able to join a session which may have influenced retention according to the participants. Also, the Exergame product itself might have influenced retention. The Exergame technology was viewed positively by the participants and the provision of feedback scores and graphs about performances appeared to stimulate interest and retention. On the occasions a technical difficulty occurred (e.g. the sensor camera not tracking the participant, updates during the sessions). These difficulties might have negatively influenced retention as older adults might have felt disappointed by this.

“The reasons (for missing sessions) were generally hospital appointments, was not well, did not feel well on that occasions. We also had issues with the computer and the package itself. That let us down itself. Maybe a session was missed by that.” – Charles, Scheme Manager.

The way the Exergames were advertised among the older adults appeared to result in different reactions from the participants. Most participants agreed that the terminology ‘exercise’ was off‐putting and that terminology such as ‘games’ or ‘fun’ would be more positively received for future recruitment.

“It's just a shame that it's called ‘exercise’ because if it was called something else it would have got more people.” – Charlotte, Scheme Manager

One SM believed that advertising using the term ‘exercise’ was not a problem and that it entails what the Exergames are about. Furthermore, the timing of the Exergames also appears to be important for the execution of the study. For example, a therapist mentioned that during the summer, many older adults would go on holiday. A SM also mentioned that it is important to check which other activities are already going on at a facility so they do not overlap.

During the execution of the Exergames in the communal lounge, the therapists also provided tea with biscuits and fruit. This was experienced, together with the relaxed atmosphere, as a facilitator for retention because it supported the creation of a social setting and social cohesion.

“And there was another guy (resident) in another group where the manager of the scheme reported he didn't used to socialize at all with the other residents. After doing the program he did so. There is a social improvements as well…Well, for the study the tea and biscuits and I know they were only little, but they do help. In real life, things that would help them comply would be giving them the option to be in a group or not [individual therapy] because everyone's different, but the majority wanted the group, so having a relaxed group environment." – Anne, Physiotherapist assistant.

“And just socially as well, it [Exergame setting] has brought people together. One lady, she was in the middle of moving into one of the schemes [Assisted Living Facilities], and she didn't know anybody so this was an excellent opportunity for her to meet other people, you know, which she probably wouldn't have done.” – Marianne, Physiotherapist assistant.

A therapist and the senior researcher also mentioned that ‘giving the older adults a voice’ was experienced as a facilitator. Explaining to older adults that they were chosen to evaluate the Exergames and that all their feedback was welcome and appreciated may also have positively influenced recruitment and retention.

## 4 DISCUSSION

The aim of this qualitative study was to explore the barriers and facilitators regarding recruitment and retention of older adults living in Assisted Living Facilities to health promoting research that aims to improve physical function by using technology. The results from this study show that SMs, therapists, researchers and older adults’ peers appear to play an important role in the recruitment and retention of older adults living in Assisted Living Facilities to health‐promoting research. Additionally, participants’ health, the technology itself and the way it was advertised and presented may have influenced recruitment and retention.

SMs of Assisted Living Facilities can play an important role in the recruitment and retention of older adults into health research. They often have a positive relationship with their residents and are able to motivate residents to participate in healthy behaviour through positive encouragement and reminders (i.e. cues to action, see Appendix 1). They appeared to be more supportive of recruitment for the study when they believed that the intervention may help the older adults and reduce their fall risk (i.e. perceived susceptibility and perceived threat according to the HBM concepts). Supportive SMs prompted older adults to attend presentations or Exergame sessions, which assisted with recruitment and retention and which may have endorsed that taking part in the study was important and worthwhile. This implies that it is worth researchers taking time to invest in gaining rapport with the SM of a facility before commencing recruitment for a study.

Therapists appeared to also influence the retention of older adults into health‐promoting research. They provided advice, praised older adults which may have improved their self‐efficacy and the therapist supervision increased the safety of participants during the intervention. Furthermore, they were able to abate tensions between older adults and they appear to be important in maintaining a comfortable atmosphere within a group which positively influences retention rates. The therapists might also have contributed to a comfortable and relaxed atmosphere by offering tea or coffee with biscuits and fruit during the sessions which also created a kind of social setting. These are all cues to action according to the HBM that may have contributed to the study recruitment and retention.

The researchers experienced that a top‐down approach can help recruitment. By first approaching the Housing Association Manager, the SMs of the Assisted Living Facilities may have been more open towards participating in the research study. Mody et al. (2008) describe in their study that care home residents are a vulnerable group and that the staff of a home might feel the need to protect the residents from exploitation (Mody et al., 2008). This could lead to scepticism of staff towards new research (Jimanez & Czaja, 2016; Mody et al., 2008). In the Exergame study, this scepticism may have been reduced due to the top‐down approach. In addition, SMs and residents were all approached not only by email or flyer but also through face‐to‐face meetings during which the Exergames and results from a previous feasibility study were presented. This approach was also acknowledged by Ford, Havstad, and Davis (2004) as having a positive impact on recruitment (Ford et al., 2004). The possible influence of the feasibility study on recruitment and retention was only briefly mentioned by the researchers as important in informing the methods of recruitment and study processes. Nonetheless, as pointed out in the reviews of McMurdo et al. (2011) and Gul and Ali (2010), a feasibility or pilot study can resolve practical issues of a large study which includes recruitment and retention (Gul & Ali, 2010; McMurdo et al., 2011). It may provide insights regarding response rates and effective recruitment strategies. Additionally, positive preliminary results can help to present the benefits for participating in the large trial.

Peer to peer support of older adults also appeared to influence recruitment and retention. Peers were found to be facilitators as well as barriers by motivating other older adults to continue to exercise by clapping and cheering but may also demotivate older adults by making negative comments or convincing them to stop. The influence of peers can be seen as contextual and environmental cues to action that affect recruitment and retention. As pointed out by Gul and Ali (2010), complex interaction of various personal and contextual factors can explain why enrolment and retention rates vary within one trial conducted at different locations in the same country (Cooley et al., 2003; Gul & Ali, 2010; Rubin et al., 2002). This explains why at some facilities peers were experienced more as a positive facilitator and at other facilities more as a barrier.

The physical and cognitive health of the residents also appeared to have an effect on the recruitment and retention of the participants. The researchers observed that the comorbid health conditions affecting the residents could at time negatively impact their ability to attend Exergame sessions due to attending clinics or requiring hospital admissions. Hall, Longhurst, and Higginson (2009) also noted the challenges of conducting research with older adults in long‐term care settings due to the range of symptoms from comorbidities and frequent acute exacerbations of conditions (Hall et al., 2009).

### 4.1 Strengths and limitations

As far as the research team is aware, this is the first analysis of the recruitment and retention strategies in a study using technology for improving physical function (Exergames) in older residents of Assisted Living Facilities. Therefore, this type of research is a valuable addition to this research field.

Furthermore, in the larger RCT study in which this sub‐study was based, the recruitment sample size was achieved. The high retention rate (87% at week 12) and recruitment across the multiple Assisted Living Facilities (*n* = 18) lead to some evidence of generalisability, an important outcome when conducting research (Stanmore et al., 2019).

However, analysis of the results of this study was conducted by two participants (the researchers) whose observations provided further insight but which might result in bias of the results. If participation and analysis is conducted by the same person, the results might be less objective. Even so, the purpose of this study is to share experiences so other researchers are aware of different types of recruitment and retention strategies. Furthermore, the two researchers analysed the results independently to limit bias.

Another limitation of this study is that no interviews were conducted with the residents regarding their experiences about recruitment and retention. Nevertheless, the researchers did investigate their experiences in the RCT by conducting focus groups at each facility at the end of the Exergame study. The findings of these focus groups will be published separately as they focused on the participant's experiences with the intervention and not specifically on recruitment and retention. The researchers have taken these experiences of the residents into account when answering the questions of the semi‐structured interviews in this study.

In conclusion, Scheme Managers, therapists, researchers and older adults’ peers can play an important role in the recruitment and retention of older adults living in Assisted Living Facilities into health research involving physical activity and technology. Also, inviting people face‐to‐face, demonstrating the Exergames during a real‐life session, showing the potential benefits by presenting results from a feasibility study and alleviating people's fears can positively influence recruitment. The results from this study can help other researcher to improve their recruitment and retention strategies. By improving recruitment and retention rates in health research among older adults, the impact of research on scientific, ethical, political and financial implications will increase. And most importantly, evidence‐based practice in care of older adults can be improved which will enhance the quality of life of older adults.

## CONFLICT OF INTERESTS

The authors have no conflict of interest to declare.

## ETHICAL APPROVAL

This research complies with the Declaration of Helsinki. Ethical approval was obtained from the NHS National Research Ethics Service, reference number 15/WA/0042.

## Appendix 1. Coding scheme

**Table:**

Main code	Sub‐code (as viewed by Therapists, Scheme Managers and Researchers)	Related to HBM concept
Residents	Characteristics Attitude (towards Exergames) Behaviour Motivation	Modifying variables, Perceived Threat, Perceived Seriousness, Perceived Susceptibility, Self‐Efficacy
Scheme Managers	Characteristics Attitude Behaviour Motivation Influence Scheme Manager‐Resident relationship	Cues to action, Perceived Barriers and Perceived Benefits
Therapists	Characteristics Attitude Behaviour Motivation Influence Trainer‐Resident relationship	Cues to action, Perceived Barriers and Perceived Benefits
Researchers	Characteristics Attitude Influence Behaviour	Cues to action, Perceived barriers and perceived benefits
Peers	Peers (influence) Dynamics and Atmosphere Housing Association	Cues to action, Perceived Barriers and Perceived Benefits
Family	Family (influence)	Cues to action, Perceived Barriers and Perceived Benefits
Scheme facilities	Scheme facilities	Cues to action, Perceived barriers and perceived benefits
Product (Exergames)	Suitability Intention to use Accessibility Influence	Cues to Action, Perceived Barriers and Perceived Benefits
Barriers	Barriers	Perceived Barriers
Facilitators	Facilitators	Perceived Benefits, Cues to Action
Benefits	Physical Mental Social Independence	Perceived Benefits
Recruitment	Recruitment	Perceived Barriers and Perceived Benefits
Adherence	Withdrawal Reasons for absence	Perceived Barriers and Perceived Benefits
